# Towards “Born-Accessible” Educational Publishing

**DOI:** 10.1007/s12109-022-09922-0

**Published:** 2022-11-07

**Authors:** Agata Mrva-Montoya

**Affiliations:** Discipline of Media and Communications, The University of Sydney, Camperdown, NSW 2006 Australia

**Keywords:** Educational publishing, Accessibility implementation, Print disability, Vision impairment

## Abstract

This paper reports on how accessibility is being slowly implemented in the current editorial and production workflows of Australian educational publishers. The findings follow from an online questionnaire commissioned by the Australian Publishers Association completed by 65 educational publishers. The paper shows that many publishers have started working on accessibility implementation, but some of them are still at the scoping stage. While many of the participants believe that the quality of “born-accessible” publication is better for all users, they are concerned about the amount of work and financial cost involved. Overall, publishers understand the need for accessibility implementation, but require further practical support and training. Publishers are also interested in working out the best workflows, timing and processes, and most cost-effective way of implementing accessibility.

## Introduction

Changing legal requirements, and growing industry interest all point to accessibility’s urgency and importance, however studies from Australia [1], Canada [2] and the European Union (EU) [3] indicate that knowledge and skills remain a challenge for publishers. Already in 2005, Frederick Bowes called on “publishers to develop and implement informed operating policies and protocols that assure that on an ongoing basis its products and services meet applicable accessibility requirements and thus can fully compete in an increasingly demanding marketplace” [4]. The commercial logic is compelling: if inclusive publishing increases the number of students able to access (that is, *use*) textbooks, it also increases potential sales. In other words, creating accessible educational resources makes good business sense, opening opportunities to serve a substantial, under-serviced market segment and helping build publishing industry capability and resilience. The critical question remains, however, what is the marginal cost of every additional user, which may explain why the decisions about investments in changing publishing processes happen at the margin and why change is somewhat fragmented.

In 2015, Australia ratified its adherence to the 2013 Marrakesh Treaty to Facilitate Access to Published Works for Persons Who Are Blind, Visually Impaired or Otherwise Print Disabled [5] and in 2016, revised its national (government) procurement rules, requiring public libraries and educational institutions to procure digital products, services and content that meet accessibility requirements. Following this, the NSW Department of Education, along with other state school systems, began reviewing their accessibility implementations and updating their procurement procedures [1]. In short, educational publishers that fail to comply with incoming requirements, will at some point become unable to sell their resources.

Further, in 2017, Australia amended its Copyright Act 1968 to legally buttress access to copyrighted material for persons with a disability and allowing producers to convert published materials (including textbooks) into accessible formats. Nonetheless, because current conversion processes remain time and resource intensive [6], resulting delays in receiving suitable class materials continue to constrain educational outcomes for students with print disabilities, creating and perpetuating disadvantage [7].

Given these rafts of changes and their surrounding issues, the need to further research implementation frameworks and develop practical planning, execution and evaluation tools, is ever more urgent, and hence, one key question presses this project forward: What is the most workable path for publishers to meet their obligations within a heterogenous industry where commitment to change, and resource/capacity for change vary significantly.? Answering this, entails understanding:

- current states of accessibility implementation among educational publishers
- types of software and other tools used at different stages of the publishing process
- supports needed to embed accessibility policies and inclusive publishing workflows.

Beginning with an outline of this project’s background and a review of available research, this paper then presents quantitative and qualitative data gathered from a recent online survey of educational publishers. Subsequent discussion examines key details regarding respondents’ accessibility implementations, taking in: methods of approach, organizational leadership, activities, challenges and perceived knowledge and skills gaps. It concludes with a look to the future and several recommendations for the Australian Publishers Association (APA). Afterward, it attends to the study’s limitations and considerations for further research.

## Background

Interest in accessibility implementation in the publishing industry is part of a broader accessibility revolution sweeping society that is also flowing into many fields of scholarship and industry. The genesis of this global cultural and societal phenomenon is closely tied to the 1948 Universal Declaration of Human Rights [8] and the 2006 UN Convention on the Rights of Persons with Disabilities [9]. These international instruments have influenced national legislative, administrative and judicial practices around the world and are transforming the way books are published. The Marrakesh Treaty was a pivotal point in the challenge of improving access to books for persons with print disabilities. Apart from globally facilitating access to existing libraries of accessible content, the Marrakesh Treaty also heightened interest in accessibility implementation in the publishing industry.

In 2019, the EU’s European Accessibility Act (EAA) [10] made adopting inclusive publishing practices more urgent, by requiring member states to implement it by 28 June 2022, with enforcement slated to start on 28 June 2025. In contrast to the exception-based Marrakesh Treaty, this EU law requires publishers to produce digital publications in accessible formats for the European market, and the entire supply chain to deliver content through accessible services. While the directive has unavoidable implications for European publishers, it also affects any non-European organization seeking to sell books to European markets.

Several research projects have investigated the accessibility implementation in the publishing industry to date. The UK-based ASPIRE project reviewed the state of e-book accessibility in 2016 [11] and the state of “accessibility information” in relation to e-books and platforms in 2018 [12]. The global DAISY Consortium has periodically surveyed publishers since 2018, but its results inevitably skew towards publishers with an active interest in inclusive publishing. DAISY’s 2020 survey revealed a promising trend towards awareness building and born-accessible content creation, and widespread adoption of the app Ace by DAISY for testing. At the same time, the cost and time required to produce good quality alt text and implement other accessibility related practices, especially in math, chemistry and scientific materials were reported as key challenges [13].

The research project carried out in Canada by the Association of Canadian Publishers and eBOUND Canada and published in 2020 focused on three areas: reviewing Canada’s English-speaking landscape and potential publishing market for accessible digital books; investigating standards and certification programs; and developing industry awareness and training strategies in relation to accessible books [2]. Based on surveys, focus groups and interviews of people with print disabilities, publishers, librarians, book distributors, alternative format suppliers and leaders in accessibility initiatives, the final report overviewed Canada’s accessible e-books and audiobooks supply chain. The study’s focus on publisher perspectives aimed to identify and offer accessibility-related recommendations regarding: “barriers to production, distribution and discovery … best practices for marketing and selling … [and] market-led [creation] incentives”. Canadian publishers reported a lack of accessibility awareness and various production, distribution, discoverability and cost concerns, making particular reference to highly illustrated books [2]. A subset of this report’s extensive list of recommendations is particularly relevant to publishers, focusing on the need for more education and training in creation of alt text, accessible e-book files and workflows used to produce them, and the need to carry out accessibility audits of files, workflows and websites [2].

A research project carried out in Australia in 2020 [1] focused on understanding what publishers and alternative content producers were doing in terms of producing accessible content, their motivations and challenges to lessen the duplication of effort in the short term, and transform the production of accessible content in the long term. The findings from the survey of publishers showed that despite much goodwill and the fact that digital book production was almost the norm, accessible production was not. Publishers articulated ethical, legal and creative motivations to produce accessible e-books, and saw the return on investment to be of lesser importance. As in Canada, skills and knowledge deficits and limited awareness were cited as key barriers and challenges to accessible e-book production. This research noted several opportunities to explore born-accessible content production, along with the suggestion that supply chain stakeholders ought to collaborate more [1]. The second survey aimed at staff in disability organizations, alternative format providers and educational institution disability support services sections focused on accessible-format conversion processes and key challenges. Its primary recommendation was for publishers to respond to requests more quickly and provide updates on their processing, fast-track access to suitable files, such as Adobe InDesign, Illustrator, EPUB, Microsoft Word or editable PDFs (free of DRM restrictions or watermarks). It also recommended that publishers have on their websites clearly defined and accessible content policy requests and procedures—which seems an easily attainable goal [6].

Several more recent industry surveys have also been carried out in Europe. A Supporting Inclusive Digital Publishing through Training (SIDPT) investigation surveyed the current state of accessibility implementation and industry training readiness to support and develop the Inclusive Publishing in Practice platform (see Braillenet [3] and [14]). In 2021, the DAISY Consortium investigated EU member implementation preparations for the EAA, including the extent to which they had engaged government ministries and stakeholder platforms. Consistent with other surveys, this effort identified awareness, training and clarity shortfalls [15]. In early 2022, the UK’s Accessibility Action Group launched a survey seeking to monitor progress and identify “gaps in solutions, knowledge resources and guidance” [16].

The above investigations informed the questionnaire design of this project, with adaptations to suit the specifics of Australia’s educational publishing sector. Unsurprisingly, the number of educational publishers in Australia is relatively modest. At the time of writing, of the 87 APA member organizations producing substantive materials for the education sector, fewer nominated educational publishing as their major revenue source (namely, 10 in scholarly and journal publishing, 27 in school educational publishing and 12 in tertiary and professional publishing) [17]. While not all educational publishers or educational technology companies in Australia are members of the APA, most key players are. APA members range from small to large, local to multinational, start-up to well-established and employ a variety of business models, and this diversity was reflected in this survey’s responses.

## Methods

This paper reports findings from a survey within a broader research enquiry into accessibility implementation in editorial and production workflows of educational publishers. The survey’s instrument proposed a set of closed and open-ended questions designed to reveal current publishing practices, software usage, production outputs, knowledge and skill levels and the industry needs to support the production of born-accessible educational materials. Of further and particular interest, was understanding the nature and extent of staff accessibility awareness over a variety of working roles and functions.

An invitation to participate was circulated directly via email to all members of the APA’s Schools Educational Publishers Committee and indirectly via newsletter to the wider APA membership. A link to the questionnaire was also shared using social media, including via LinkedIn and Twitter. To cover the range of relevant roles—in acquisitions, editorial, rights and permissions, production, marketing and management—all staff of educational publishers were invited to participate, anonymously and voluntarily.

The questionnaire instrument was created using Qualtrics and structured to capture limited demographic information that might prove material to understanding a possible variety of approaches. Respondents were asked for example, to note the size and nature of their organization, addressable market segments (primary and secondary school, higher education, vocational and educational training) and individual function or role.

Respondents were asked to begin by providing informed consent, and to finish by supplying an email address if they wished to be notified of results or would consent to a follow-up interview. The latter option was disaggregated from the responses to the main questionnaire.

After testing with a small sample of publishing professionals, the questionnaire was disseminated as noted above, and available for responses from 15 April to 31 May 2022. The data was subject to qualitative (free text) analysis using Microsoft Excel and quantitative analysis (multiple choice questions, as well as re-coded free text responses) using a combination of SPSS and Microsoft Excel.

## Findings

**Fig. 1:**
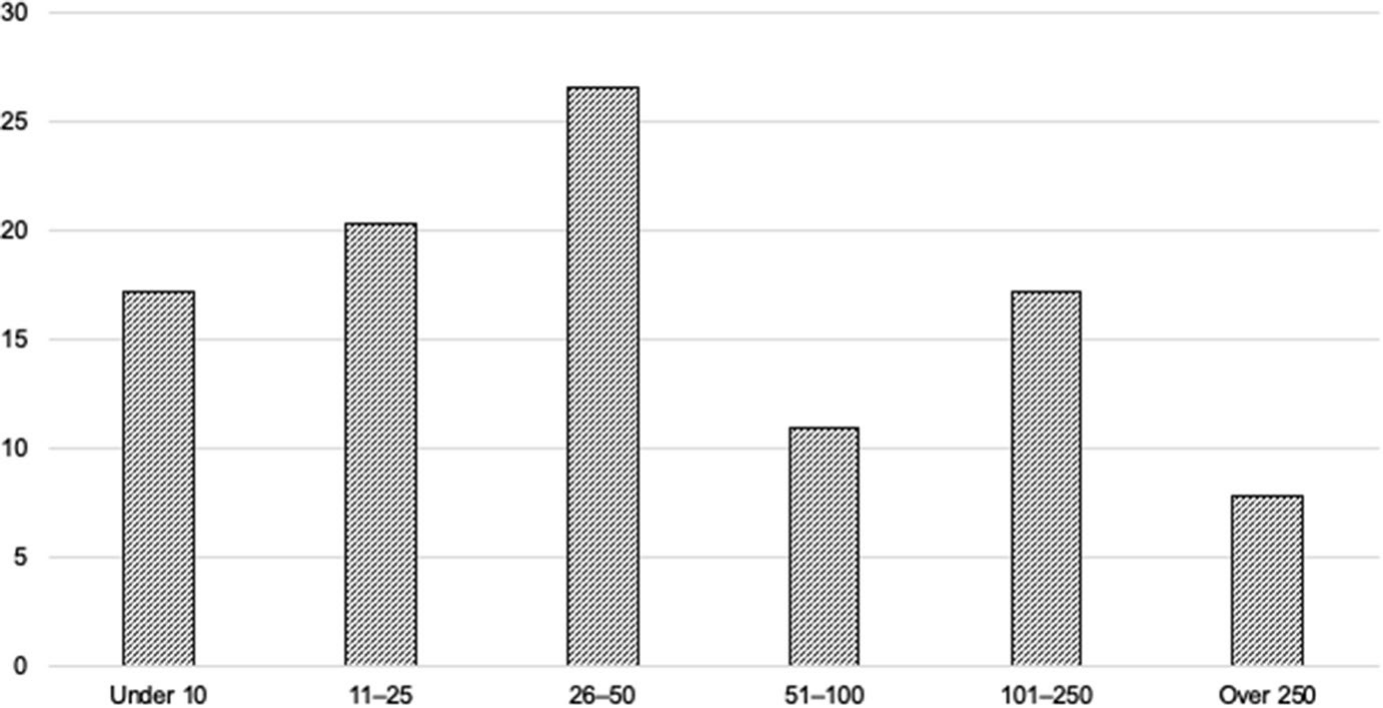
Publishers’ size by number of employees

The survey of educational publishers received 65 responses from staff in management, editorial, acquisition, commissioning, project management and other areas of business. These respondents predominantly represented independent publishers (54%), then global publishing groups (42%), and individual professionals from an academic publisher, a government publisher and an education technology company. The entities represented ranged in size from several having fewer than 10 employees, to one with 500 (see Fig. 1).

The survey permitted multiple choice responses to the question of organizational target market segments, and 80% of respondents said they publish for primary schools, 51% for secondary schools, 42% for higher education and 26% for vocational and educational training.

Key resources and services produced included: teacher/lecturer support material (89%), textbooks (87%), tests and assessments (69%), e-learning assets (66%), other student resources (52%), student study guides (45%), professional development resources and events (42%), learning apps (39%), other educator resources (23%), parent resources (21%), games (19%) and others.

Print remained the substantively dominant output, with over 98% of publishers releasing products and resources in that medium, however, just one respondent cited print as their organization’s *only* output. Regarding digital, PDF was the most popular format (76%), followed by HTML (53%), e-book app (37%), EPUB3 file (34%) and 21% of publishers still produce EPUB2 files. Many publishers also produce videos (55%), webinars (55%) and events (32%). Other production formats noted were: XML (21%), other online formats (18%), CDs and DVDs (11%), USBs (10%), podcasts and audio resources.

The publishers reported using a staggering variety of software packages across the various stages of the publishing process, which was predominantly carried out inhouse.

At the acquisition and product conception stage, Microsoft Office was considered key, followed by Adobe Acrobat and Adobe Creative Cloud. Respondents also noted using different systems for more specific functions in this stage however, namely for: project and data management, file sharing and collaboration, market research, user testing, cloud-based e-signature services, non-Adobe graphics and web design, software development and simulation, and (Zoom) video conferencing.

Authoring processes and content development were managed using Microsoft Office (mainly Word), followed by Adobe Acrobat, Adobe Creative Cloud and Google Docs. Three respondents mentioned using a “proprietary XHTML authoring/content development/editing/proofing/production platform (vendor-owned)”, and another two noted a third-party platform for digital content. Again, respondents also mentioned a variety of more function-specific platforms in use, in relation to e-learning, digital assessment solutions, video editing, file sharing and FTP transfer, project management, non-Adobe PDF editing, and software development and simulators.

Workflows, projects and business processes were largely managed using Microsoft Office, followed by the Google Suite, but again, other platforms were useful, for example, in relation to time tracking and file sharing.

Design and layout were mostly reported as involving Adobe Creative Cloud: especially InDesign but also Illustrator, Photoshop and Acrobat. Two respondents noted using third-party platforms for digital, and one, the aforementioned vendor-supplied XHTML platform. One respondent reported entirely outsourcing design and layout.

For proofing, most respondents reported using Adobe Acrobat, followed by Microsoft Office applications—typically Word and occasionally Excel. Again, respondents also noted that more specific tasks and functions required correspondingly specific platforms and applications, either relating to or involving: Adobe software (InDesign/InCopy), HTML editors in different online platforms as well as e-book specific platforms; learning, assessments, and training management tools, video editing platforms, and a proprietary XHTML platform mentioned above. Two respondents reported that they outsourced proofing.

In quality assurance and testing, respondents reported using Adobe Acrobat, Microsoft Word, EPUB checker and Ace by DAISY. Respondents also referenced a variety of third-party e-book and digital resource platforms, app development software, video editing platforms, digital assessment solutions and collaboration tools. One respondent outsourced this stage.

Regarding sales and marketing, a variety of customer relationship management (CRM) systems were in high use. Respondents also referenced Microsoft Office, Adobe Creative Cloud, Google Apps, online stores (including one digital content specific distribution platform), social media and collaboration platforms. One respondent noted using an AI-driven marketing tool.

To distribute products, respondents relied on their own organizational websites as well as more specialist software and systems relating to e-learning, accounting, customer relations and business management—where again, Microsoft Office remained in use.

Among the 42 respondents who responded to the question about the views of their organisation with “regards” to accessible publishing (respondents were asked to choose all answers that apply):

- 64% considered that meeting accessibility requirements would improve quality and user experience across all their digital products
- 60% considered it to be a social and moral responsibility
- 40% were concerned about the amount of work and financial cost involved
- 29% were aware of accessibility requirements but had not yet taken steps to integrate them into publication workflows
- 21% said they wanted to produce accessible digital publications, but were not sure how to do so
- 10% claimed to be unfamiliar or unsure
- 7% had no capacity (knowledge, resources or otherwise) to initiate change
- 7% saw no financial or other benefit for their business
- 5% were worried that it would erode publications quality
- 5% viewed it to be the government’s responsibility.

On the question of progress, and more specifically, whether organizational action had begun on accessibility implementation, a majority of respondents (59% of n = 41) reported having started to integrate accessibility into their publishing workflow, while 17% had not and 24% remained unsure. Table 1 shows that larger companies were far more likely than smaller ones to have started such engagements (n = 40).

**Table 1:** Accessibility implementation engagements by organizational size

Org. size (by no. employees)	Yes (%)	No (%)	I don’t know
Under 10	28.60	71.40	–
11–25	42.90	14.30	42.90
26–50	72.70	–	27.30
51–100	60.00	20.00	20.00
101–250	77.80	–	22.20
Over 250	100.00	–	–
Total	60.00	17.50	22.50

Respondents (n = 41) nominated a variety of ways their entities were approaching accessibility implementation:

- 32% had tasked oversight to an individual or team
- 27% monitored their products for compliance on a regular basis
- 24% provided awareness training to their employees
- 20% embedded accessibility into product conception and authoring processes
- 17% regarded accessibility as integral to their policies
- 17% employed service providers and freelancers to comply with requirements
- 12% always checked compliance before publishing
- 10% provided skills training to employees
- 5% involved people with print disabilities in design and development processes.

At the same time, 32% of respondents had not yet taken measures to make their publications accessible and 17% were not sure. In one organisation, “Accessibility is seen as critical and is being implemented but there is a huge cost imperative, and we are still trying to work out the best workflows and timing and processes and most cost-effective way of implementing it.” In contrast, another respondent wrote that, “In previous years, accessibility was an afterthought, something that was added retrospectively and content had to be remediated. Now, accessibility is designed well upstream from the point of content creation. It was originally something only for digital products, but now we are considering accessibility requirements in print too.”

The process has been driven by an accessibility/diversity and inclusion working party/task force at four publishers (out of 12 respondents), in two by production teams, in another two by learning designers, in one by editorial manager, and in one by the UK content team. In one company a mandatory accessibility awareness and training program was globally rolled out for all staff. Five of the respondents mentioned the support of senior management.

Five respondents (n = 14) reported organizational embedding of accessibility at the production stage. Two embedded it (especially for digital products) at the point of content creation, and another two claimed to be overall actively working towards inclusive publishing practices. Four respondents were investigating accessibility implementation, but said they needed more training and advice. One respondent noted that organizational engagement with accessibility was limited to providing files to a “university support team”.

Publishers (n = 31) were more confident implementing some accessibility-related tasks than others, and were most comfortable with:

- using legible typefaces (63%)
- accounting for readability principles in layouts (61%)
- structuring texts with style sheets (55%)
- seeing accessibility as part of editing and proofreading (53%)
- using sufficiently contrasting colours (53%)
- adding text alternatives to non-textual content (42%).

Barely a quarter of publishers reported running accessibility quality assurance (26%), adding accessibility metadata (26%), or making multimedia accessible for students who are blind (26%). The inclusion of alternative text is one of the key elements of making content accessible and typically authors (44%), development editors (44%) or subject specialists (31%) are responsible for its creation, with some of the 16 publishers outsourcing the task to local (18%) or overseas vendors (25%). Editors (80%) or proofreaders (53%) are typically responsible for checking the alternative text provided.

Fewer than half of respondents (38%, n = 38) had undertaken accessibility production training, whether for print, digital or both. Those who had, did so via online webinars (for example, as provided by the DAISY Consortium), internal online workshops and peer-to-peer sharing. Respondents generally indicated clear needs for:

- increased expertise and capacity to enable further accessibility improvement
- unified, streamlined and simplified standards, contacts, processes and services
- instructional materials designed “to guide scoping and development decisions”.

As one respondent added, “We are concerned about finding the best training to help us with implementation. We would like to know about expert services that can help guide internal teams, or even where we can outsource certain tasks like alt-text development.”

In terms of training required, there is a clear appetite for practical sessions with accessibility “best practice”. Other specific training needs which topped the list include: images and text alternatives (illustrations, maps, infographics, graphs, etc.), tables (format best practice, alternative text, etc.), graphic and layout (contrast, use of colour, responsive design, etc.), interactive elements. There is less interest in training on accessibility policy, business context and legislation, which shows that publishers understand the need for accessibility implementation, but need further practical support.

## Discussion

Despite having their progress slowed by the COVID-19 pandemic [18], a notably higher proportion of respondents (almost 60%) indicated engagement with accessibility compared with Australia’s (40%) broader publishing industry result in 2020 [1]. Overall, this is not surprising, given educational publishers’ stronger legal, moral and commercial imperatives. While many of the participants believed the quality of *born-accessible* publication to be better for all users, they remained concerned about the volume of work required and financial cost involved. The question of cost was also raised by publishers in Canada.

A small proportion of respondents reported having either no capacity for accessibility or seeing no benefits in its implementation. Few remained concerned that implementing accessibility might adversely affect publication quality. This suggests a certain lack of understanding of the principles of inclusive design, which accrue positively beyond the needs of students with print disabilities.

This survey found that larger publishers were more likely than smaller ones to already be working towards producing accessible materials. Some respondents were uncertain about their organization’s progress in starting work. If this was because they were not directly involved in the process, that may also point to poor company-wide policy communication, especially in smaller organizations, but in any case, it indicates a lack of accessibility engagement. This differs from the findings of the 2020 survey, where publishers of all sizes have been able to produce accessible content. As educational publications are generally more complex than other kinds of text, they require greater investments to make them accessible, and therefore, the need for greater human, organizational and financial resources is not surprising.

Embedding accessibility in the production stage is somewhat more common than adopting inclusive publishing practices, but it could be a transitional stage as further two respondents reported exploring embedding accessibility into the whole publishing process. As some organizations are exploring this more positive direction however, it seems plausible to expect that in the future, this current lag may prove to have been a transitional delay. At some organisations the process is being driven by teams tasked specifically with focusing on accessibility or diversity and inclusion. In others, it is managed by functional teams (such as production or learning design). A whole-company approach is rare, with only one respondent reporting an all-staff mandatory training on accessibility awareness.

Respondents commonly considered accessibility in content structure, graphic design, alternative text descriptions, editing and proofreading. Fewer reported thinking about accessibility in terms of metadata, multimedia and quality assurance. This latter point is inconsistent with the DAISY Consortium’s 2020 report of widespread adoption for Ace by DAISY app for testing. Even so, neither DAISY’s app, nor accessibility checkers such as those available in Adobe PDF and Microsoft Word obviate the need for manual accessibility review and testing of, but this step was missing from most respondents’ workflows. It is also worth noting here, that Canadian work exploring certification program feasibility endorsed Benetech’s Global Certified Accessible program (GCA) as being able to “increase publisher awareness, confidence and capability” [2].

Interestingly, educational publishers seem to rely less on outsourcing production than the industry average in Australia, noted in the 2020 survey. Respondents reported using a remarkable variety of software systems, packages and platforms across the various publishing processes, producing a corresponding diversity of educational resources and formats. Adobe InDesign remains key to the sector. Unfortunately, EPUB files created using Adobe InDesign lack several important accessibility features, including: accessibility metadata, page lists and number locators, ARIA roles, ability to include extended image descriptions, add structured code and language tagging. Moreover, it is difficult to create sections and landmarks in InDesign, and the resulting file contains needlessly complex code [19]. Remediating EPUB files created using Adobe InDesign is thus necessary, but demands extra work and cost, in addition to the further expense in creating alternative text.

While publishing workflows are already highly digitized, print remains the most common production output. Still, almost all respondents reported producing resources in digital formats. The popularity of PDFs is unsurprising but ensuring this format’s accessibility remains challenging. Given the EPUB3 format was released over a decade ago (in 2011) and that it is natively more accessible than its predecessor—offering richer navigation, being human- and machine-readable and containing support for multimedia and MathML—the ongoing prevalence of EPUB2 is surprising, and concerning [20].

Respondents demonstrated healthy appetites to learn about best practice, workflows, timing and processes, in order to cost-effectively implement accessibility. They were less interested in undertaking training in matters of policy, business context and legislation, which publishers no doubt more broadly understand. In sum, the standout training need in relation to accessibility implementation, is for more practical support.

## Conclusions

A range of contemporary global and local technological developments, cultural emphases, legislative enactments and industry commitments point to a continuing intense focus on the need to implement more inclusive publishing practices. This project’s findings reveal a sector with complex digital workflows, which is still very much in transition toward making “born-accessible” publishing a reality, with larger publishers reporting being further along the path toward producing accessible publications.

Educational publishers in Australia are at least aware of, if not engaged at some level with accessibility implementation, and generally supportive of the idea that natively accessible educational resources would be better for all. Nonetheless publishers have caveats, or at least questions, concerning required volume of work and financial costs. At present, publishers typically adopt accessibility at the production stage, where it is somewhat hampered by (among other things) publisher reliance on Adobe InDesign, with its inadequate support for accessibility. Although publishers have been focusing on ensuring the correct content structure, accessible graphic design, and the inclusion of alternative text descriptions, few have addressed the automatic and manual quality assurance processes needed to check accessibility compliance.

While far more resources on inclusive publishing practices are available now than in 2020, educational publishers in Australia lack structured experience, tailored guidelines and practical training on best workflows, timing, and cost-effective means of delivery. Here, perhaps the industry’s peak body, the APA—which has already committed to supporting “educational publishers to meet the mandatory requirements for accessible learning materials” [21] —might further prioritize equitable sector-wide capacity-building, taking care not to neglect smaller publishers. There is a clear need to continue the work of the Australian Inclusive Publishing Initiative [22] in leading the education of the publishing sector, tracking progress in accessibility implementation, and working with other stakeholders in the book supply chain. With this and other accessible solutions in sight, in spite of undoubtable challenges—including that many educational publishers have a long way to go—it seems safe to predict that, in the not-too-distant future, “born-accessible” educational resources will become the publishing norm.

## Limitations

Care should be taken not to apply these results overly strictly as representative of Australia’s entire education publishing sector due to the possible introduction of respondent self-selection bias in the project methodology, and the instrument’s low sample size. Moreover, while an online questionnaire is a useful tool for gathering preliminary data, it does not allow fuller exploration that would deliver more nuanced reasoning, attitudes and opinions. For example, it would be interesting to know why publishers are still producing EPUB2 files and what processes they use to remediate accessibility in files produced using Adobe InDesign. Further qualitative research incorporating person-to-person interviews could thus investigate the motivations, challenges and practices of individual publishers in more detail, as well as better explore appetites and feasibilities for an industry-endorsed certification program.
